# The Pathogenesis of Very Long-Chain Acyl-CoA Dehydrogenase Deficiency

**DOI:** 10.3390/biom15030416

**Published:** 2025-03-14

**Authors:** Shashwat Sharma, Matthew McKenzie

**Affiliations:** 1 School of Life and Environmental Sciences, Faculty of Science, Engineering and Built Environment, Deakin University, 75 Pigdons Road, Waurn Ponds, VIC 3216, Australia; sharmashas@deakin.edu.au; 2 Institute for Physical Activity and Nutrition, Deakin University, 75 Pigdons Road, Waurn Ponds, VIC 3216, Australia

**Keywords:** VLCADD, very long-chain acyl-CoA dehydrogenase deficiency, VLCAD, very long-chain acyl-CoA dehydrogenase, FAO, fatty acid β-oxidation, FAOD, fatty acid oxidation disorder, oxidative phosphorylation, OXPHOS

## Abstract

Living systems require energy to maintain their existence and perform tasks such as cell division. This energy is stored in several molecular forms in nature, specifically lipids, carbohydrates, and amino acids. At a cellular level, energy is extracted from these complex molecules and transferred to adenosine triphosphate (ATP) in the cytoplasm and mitochondria. Within the mitochondria, fatty acid β-oxidation (FAO) and oxidative phosphorylation (OXPHOS) are crucial metabolic processes involved in generating ATP, with defects in these pathways causing mitochondrial disease. Very long-chain acyl-CoA dehydrogenase deficiency (VLCADD) is a fatty acid β-oxidation disorder (FAOD) affecting 1 to 2 individuals per 100,000. Similar to other mitochondrial disorders, there is no cure for VLCADD, with symptomatic treatment comprising dietary management and supplementation with medium-chain fatty acids to bypass the enzyme deficiency. While this addresses the primary defect in VLCADD, there is growing evidence that other aspects of mitochondrial function are also affected in VLCADD, including secondary defects in OXPHOS function. Here, we review our current understanding of VLCADD with a focus on the associated biochemical and molecular defects that can disrupt multiple aspects of mitochondrial function. We describe the interactions between FAO proteins and the OXPHOS complexes and how these interactions are critical for maintaining the activity of both metabolic pathways. In particular, we describe what is now known about the protein–protein interactions between VLCAD and the OXPHOS supercomplex and how their disruption contributes to overall VLCADD pathogenesis.

## 1. Mitochondrial Metabolism

Mitochondria are responsible for producing most of the ATP required for life processes, with daily production of ATP almost equal to the weight of a human body (approximately 72 kg) [1]. The oxidation of sugars, fats, and proteins by mitochondria results in the generation of two important reducing equivalents, NADH and FADH_2_. These two molecules are derived from a number of different sources, including glycolysis (NADH), the tricarboxylic acid (TCA) cycle (NADH and FADH_2_), and mitochondrial fatty acid β-oxidation (FAO) (NADH and FADH_2_). Electrons from NADH and FADH_2_ are transferred to the electron transport chain (ETC) in the inner mitochondrial membrane, which drives proton (H^+^) pumping to generate a proton motive force (pmf). Lastly, ATP synthase utilizes the pmf to drive the generation of ATP from ADP and inorganic phosphate (P_i_). This combination of substrate oxidation, electron transfer, and ATP generation is known as oxidative phosphorylation (OXPHOS).

## 2. Oxidative Phosphorylation

OXPHOS is performed by five complexes imbedded in the inner mitochondrial membrane; complex I (CI, NADH:ubiquinone oxidoreductase), complex II (CII, succinate:ubiquinone oxidoreductase), complex III (CIII, ubiquinol:ferricytochrome *c* oxidoreductase), complex IV (CIV, cytochrome *c* oxidase), and complex V (CV, F_1_F_o_-ATP synthetase).

NADH and FADH_2_ are oxidized by CI and CII, respectively, with the available electrons reducing ubiquinone (Q) to ubiquinol (QH_2_). QH_2_ is oxidized by CIII, which results in the reduction of cytochrome *c*. Cytochrome *c* is then oxidized by CIV, reducing molecular oxygen (O_2_) to water. This flow of electrons drives the pumping of protons by complexes I, III, and IV from the mitochondrial matrix into the intermembrane space, creating an electrochemical gradient across the inner membrane (the mitochondrial proton motive force, pmf). The pmf is used to drive CV activity, which phosphorylates ADP to generate ATP.

To enhance electron transfer between the OXPHOS complexes, and/or facilitate efficient packing in the inner membrane, high-order interactions between the OXPHOS complexes have been identified [2]. In particular, CI, CIII, and CIV form structures termed OXPHOS supercomplexes or ‘respirasomes’ [2,3]. These supercomplexes can exist in varying combinations but are found predominantly in CICIII_2_CIV, CICIII_2_, or CIII_2_CIV_4_ forms [4]. The specific roles played by these supercomplexes remain somewhat elusive; substrate channeling to regulate electron transfer efficiency (and potentially reactive oxygen species generation) between the OXPHOS complexes was initially proposed but is not supported by more recent biochemical data [5]. Alternatively, the supercomplexes may play a role in regulating the stability and/or assembly of the OXPHOS complexes, with supercomplex assembly occurring before the completion of individual OXPHOS complex biogenesis [6].

**Figure 1:**
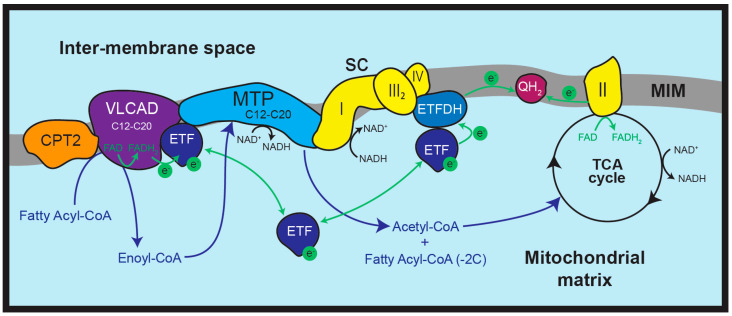
Overview of mitochondrial long-chain fatty acid β-oxidation (FAO) and interactions with OXPHOS protein complexes in mammalian mitochondria. In the mitochondrial inner membrane (MIM), very long-chain acyl-CoA dehydrogenase (VLCAD) is bound to carnitine O-palmitoyl transferase 2 (CPT2) and the mitochondrial trifunctional protein (MTP), with the MTP linked to OXPHOS complex I (I). Complex I is found in conjunction with a complex III dimer (III_2_) and complex IV (IV) to form the OXPHOS supercomplex (SC). In the initial stages of FAO, long chain fatty acyl-CoAs (C12–C20) are transported into the mitochondria through the carnitine shuttle system, which includes CPT2, and then converted into enoyl-CoAs by VLCAD dehydrogenation. Enoyl-CoAs are subsequently metabolized to acetyl-CoA (and a fatty acyl-CoA shortened by two carbons) by MTP hydration, dehydrogenation, and thiolysis. Reduced NADH is also generated during this dehydrogenation reaction. The shortened fatty acyl-CoA re-enters the FAO cycle, while acetyl-CoA is further catabolized by the tricarboxylic acid (TCA) cycle. Acetyl-CoA oxidation in the TCA cycle results in the reduction of NAD^+^ to NADH and the reduction of the FAD prosthetic group of succinate dehydrogenase (OXPHOS complex II) to FADH_2_. Complex II then reduces ubiquinone to ubiquinol (QH_2_). During FAO, the FAD prosthetic group of VLCAD is also reduced to FADH_2_, with subsequent electron (e^−^) transfer to the electron transfer flavoprotein (ETF). ETF then disassociates into the mitochondrial matrix to find the electron transfer flavoprotein-ubiquinone oxidoreductase (ETFDH), which is itself bound to complex III in the OXPHOS supercomplex. ETFDH then reduces ubiquinone to contribute to the pool of MIM ubiquinol (QH_2_). Figure based on concepts from [11,12].

Recent investigations suggest the relative abundance of specific supercomplexes depends on the tissue type as well as the in vivo metabolic needs [7,8,9]. In addition, growing evidence suggests the formation of a ‘multifunctional mitochondrial protein complex’, which includes the OPXHOS supercomplex in combination with various FAO enzymes [10,11] (Figure 1). These protein interactions will be discussed in detail below.

## 3. Mitochondrial Fatty Acid β-Oxidation (FAO)

Mitochondrial fatty acid β-oxidation (FAO) is performed by at least 20 proteins involved in the transport, activation, and oxidation of fatty acids [13]. FAO is an essential process, as a substantial amount of energy is produced from fat metabolism.

Mitochondrial FAO is responsible for metabolizing fatty acyl-CoAs with carbon chain lengths varying from small (C4–C6) to long (C12–C20). Very long-chain fatty acids (>C20) are firstly shortened by oxidation in peroxisomes prior to mitochondrial FAO [14,15,16,17,18,19]. Fatty acids circulate in the bloodstream bound to albumin, and upon entering a target cell, are transported into the mitochondria. This happens in two ways, depending upon the length of the fatty acid chain. Fatty acid chains of up to 12 carbons can be transported by passive diffusion across the mitochondrial membrane, whereas chains longer than 12 carbons require a special mechanism involving fatty acid manipulation. The fatty acid is first converted into an acyl-CoA ester by acyl-CoA synthetase. The acyl-CoA is then converted into an acylcarnitine by carnitine O-palmitoyl transferase 1 (CPT1) at the outer mitochondrial membrane. Subsequently, carnitine acylcarnitine translocase (CACT) transports this acylcarnitine across the inner mitochondrial membrane into the mitochondrial matrix. The acylcarnitine is then reverted to an acyl-CoA ester by carnitine O-palmitoyl transferase 2 (CPT2) on the matrix side of the inner membrane.

Once inside the mitochondrial matrix, the fatty acyl-CoA is oxidized via FAO in four steps; dehydrogenation (acyl-CoA → enoyl-CoA), hydration (enoyl-CoA → 3-hydroxyacyl-CoA), a second dehydrogenation (3-hydroxyacyl-CoA → 3-ketoacyl-CoA), and thiolysis (3-ketoacyl-CoA → acetyl CoA and acyl-CoA shortened by two carbon atoms) [20].

The first dehydrogenation is carried out by a group of enzymes known as acyl-CoA dehydrogenases (ACADs) [21]. Depending on the fatty acyl-CoA chain length, the enzymes involved are very long-chain acyl-CoA dehydrogenase (VLCAD; C12–C20), medium-chain acyl-CoA dehydrogenase (MCAD; C6–C12) and short-chain acyl-CoA dehydrogenase (SCAD; C4–C6). A long-chain acyl-CoA dehydrogenase (LCAD; C14–C18) also exists in mice but is almost undetectable in most FAO-dependent human tissues including heart and skeletal muscle [22,23]. However, LCAD expression has recently been detected in some human tissues, including liver, kidney, lung, and pancreas [23].

In addition, other ACADs, including ACAD9, ACAD10, and ACAD11 [24,25] have also been described. While ACAD9 has measurable FAO dehydrogenase activity, it is believed that its primary role is to assist OXPHOS complex I biogenesis as an assembly factor [26] (discussed in further detail below). Interestingly, ACAD9, ACAD10, and ACAD11 are expressed primarily in the brain [25]. While the role of fatty acid oxidation in overall brain metabolism is not completely understood, studies have shown that FAO occurs readily in astrocytes, but not in neurons [27]. However, FAO within astrocyte mitochondria is not used primarily for ATP generation, but rather the production of ketone bodies, which are subsequently utilized by neurons as a metabolite [28]. Indeed, it is now known that astrocytes provide numerous metabolites (including lactate) to support neuronal function [29]. A detailed description of brain fatty acid metabolism is out of the scope of this review; however, comprehensive reviews can be found here [30,31].

Following the first dehydrogenation, enoyl-CoA products undergo hydration, a second dehydrogenation, and thiolysis in a similar chain-length specific fashion. Enoyl-CoAs of 12 or more carbons are processed by the mitochondrial trifunctional protein (MTP), which has hydration [long-chain 2,3-enoyl-CoA hydratase (LCEH)] activity, dehydrogenation [long-chain 3-hydroxyacyl-CoA dehydrogenase (LCHAD)] activity, and thiolysis [long-chain 3-ketoacyl-CoA thiolase (LCKAT)] activity [32]. For enoyl-CoAs of 12 carbons or less, the MTP is replaced by three separate enzymes, short-chain enoyl-CoA hydratase 1 (ECHS1), 3-hydroxyacyl-CoA dehydrogenase (HADH), and 3-ketoacyl-CoA thiolase (KAT).

The fatty acyl-CoA chain is reduced by two carbons per cycle, with oxidation continuing until only two acetyl-CoA molecules (two carbons each) are obtained. Due to this continuous reduction in fatty acyl-CoA chain length, FAO is commonly described as an ‘oxidation spiral’ [13]. For each cycle of FAO, the ACADs also transfer electrons via their prosthetic FAD groups to the electron transfer flavoprotein (ETF), then the electron transfer flavoprotein–ubiquinone oxidoreductase (ETFDH), which reduces ubiquinone to ubiquinol (QH_2_). ETFDH is bound to complex III in the OXPHOS supercomplex, providing a direct source of QH_2_ for oxidation by complex III [18] (Figure 1). The second dehydrogenation reaction by MTP (LCHAD activity) or HADH also generates one molecule of reduced NADH per cycle.

Recent studies associated with ROS production add further complexity to mitochondrial fatty acid metabolism. During high energetic demand, fatty acid oxidation will increase the mitochondrial matrix pool of reduced ubiquinol and NADH. High ubiquinol levels can induce reversal of electron flow through CII, with production of oxidized ubiquinone, the reduction of fumarate to succinate, and a switch to anaplerotic and anabolic reactions within the mitochondria [33,34]. Additionally, some of the matrix pool of FAO-generated NADH may be oxidized by the nicotinamide nucleoside transhydrogenase (NNT) to maintain the cytosolic pool of NADPH to fuel free-radical scavenging enzymes and/or other biosynthetic pathways [12].

## 4. FAO Disorders

FAO produces energy in the form of ATP by catabolism of fats. As described above, many enzymes are involved in this complex process, from the transport of fatty acids into the mitochondria to their subsequent oxidation. Loss of activity of any of these enzymes can result in fatty acid β-oxidation disorders (FAOD). Presentations such as seizure, hypotonia and hypoglycemia can occur, particularly under fasting conditions or when energy demand is high, such as during exercise [13]. Skeletal muscle, being among most energy demanding tissues of the body, is commonly affected in FAOD, with symptoms including myalgia, muscle weakness, fatigue, muscle pain and rhabdomyolysis in some severe cases [13,35]. FAOD may also lead to lifelong neurological damage, and therefore have been included in various neonatal screening programs [36].

Some FAODs, such as MCAD deficiency (MCADD), can cause hypoketotic hypoglycemia during illness, fasting, exercise, or fever, and subsequently result in a life threatening coma [36]. Other disorders, including VLCADD and MTP deficiency (MTPD), are associated with hypertrophic or dilated cardiomyopathy [13,37].

Diagnosis of FAOD was initially difficult due to the highly varied symptoms, with no molecular techniques available. The introduction of Tandem Mass Spectrometry (TMS) in the late 20th century revolutionized FAOD diagnosis, and has become crucial for neonatal screening [38]. In FAOD, non-oxidized acyl-CoAs are exported back out of the mitochondria by CPT2 as acylcarnitines. Hence, accumulation of acylcarnitines is a useful marker for FAOD [39], with specific deficiencies identifiable by examination of the acylcarnitine profile. For instance, acylcarnitines longer than C14, and shorter than C20 suggest VLCADD [40]. This acylcarnitine profiling is usually followed by genetic confirmation upon signs of fatty acid oxidation defects [41].

Acylcarnitine profiles can then direct further investigation via patient blood analyses, rather than using more invasive diagnostic techniques. Human lymphocytes can be used to rapidly diagnose FAOD, as these cells express all enzymes involved in FAO [42]. Moreover, functional assessment of β-oxidation in cultured fibroblasts, and/or direct assessment of enzyme activity using fibroblasts or lymphocytes, can also be performed [42,43].

## 5. VLCAD Deficiency (VLCADD)

VLCAD is situated in the inner mitochondrial membrane, forming a 150 kDa homodimer [44]. Participating in the first dehydrogenation step of FAO, VLCAD is active for fatty acyl-CoAs containing 12 to 24 carbons, with optimal specificity for 16 carbon fatty acyl-CoAs [45]. However, in vivo VLCAD is exposed to fatty acyl-CoAs of a maximum C20 length only. VLCAD oxidizes the β-carbon of the fatty acyl-CoA, with a reduction of its co-factor FADH to FADH_2_ (Figure 1). Human VLCAD, similar to other FAO enzymes, is abundantly active in liver, heart, and skeletal muscle [46].

VLCADD was first described in 1993 in a two-day-old girl with ventricular fibrillation and abnormal carnitine metabolism after nocturnal feeding refusal [47]. Of all the long-chain FAODs, including MTPD and CPT deficiency (CPTD), VLCADD is the most common among most populations [48]. It is responsible for significant morbidity and mortality among newborns, early teens, and adults [49]. VLCADD is caused by autosomal recessive mutations in *ACADVL*, located at 17p13.1 (OMIM#201475), with an incidence of 1 to 2 per 100,000 births. However, the true frequency may be different, as patients can be non-symptomatic and/or have false negative (or false positive) diagnoses [48]. The phenotype–genotype relationship of VLCADD has also been difficult to resolve, possibly due to the redundancy provided by other FAO dehydrogenases for fatty acid oxidation, such as ACAD9 and MCAD, with some overlapping of substrate specificity ranging from 10 to 22 carbon chain lengths [42,45,50] (although some ACADs do exhibit tissue-specific expression and optimal activity for particular chain lengths [24,45]).

The severest VLCADD symptoms tend to occur in individuals with null *ACADVL* mutations that result in the complete absence of VLCAD protein expression. Conversely, milder forms of VLCADD are usually associated with *ACADVL* missense mutations [43]. Several *ACADVL* mutations have been described, with c.848T>C being the most common variant [51] (Table 1).

VLCADD can be divided into two major categories depending on its presentation: early onset (severe), and later onset (mild). Early onset VLCADD presents as recurrent cardiomyopathy, acidosis, hepatic dysfunction, and hypoglycemia in early stages of life. Patients who survive early onset symptoms face a 75% mortality rate within the first few months [43]. Childhood onset VLCADD, which is milder than early onset, can present with hypoketotic hypoglycemia with lower risk of cardiomyopathy. Association with rhabdomyolytic events in later stages of childhood have also been described [65].

### VLCADD Treatment

For patients with VLCADD, a diet containing high carbohydrates is recommended in case of rhabdomyolytic episodes [66]. Diet supplementation with medium-chain triglycerides such as Triheptanoin are also being used to bypass the deficiency in long-chain fatty acid oxidation [67]. Triheptanoin is a 7-carbon chain triglyceride that is FDA-approved for long-chain FAOD treatment. However, medium-chain supplements such as Triheptanoin are not completely effective for VLCADD patients, as inconsistent outcomes have been reported [68]. Furthermore, higher oxidative stress has been reported in VLCADD mice with medium-chain supplementation [69].

Recent studies have examined the stimulation of mitochondrial biogenesis as a potential treatment strategy for VLCADD. Agonists of peroxisome proliferator-activated receptors (PPARs), transcription factors involved in stimulating mitochondrial biogenesis (including FAO and OXPHOS enzymes), have improved mitochondrial function in VLCADD patient fibroblasts [70,71,72]. REN001, a PPARδ agonist, improved mitochondrial oxygen consumption and ATP production in vitro. Notably, this drug significantly increased *ACADVL* gene expression in mild mutations with some residual enzyme activity, but only a small increase in VLCAD protein and enzyme activity were observed [71].

Bezafibrate, a pan-PPAR agonist, has been tested in both VLCADD patients and isolated VLCADD patient cell lines. VLCADD cells showed improvement in mitochondrial metabolism with restoration of VLCAD activity and reduction in acylcarnitine accumulation [73]. Additionally, bezafibrate reduced hospitalizations associated with myopathic attacks in five VLCADD patients [74]. However, patients with mitochondrial myopathy carrying the m.3243A>G MTTL1 mutation showed increased serum biomarkers of mitochondrial disease and dysregulation of fatty acid and amino acid metabolism following bezafibrate treatment [72]. In addition, bezafibrate was shown to increase oxidative stress in VLCADD patient fibroblasts, reducing viability [75]. Thus, bezafibrate treatment of mitochondrial disorders (including VLCADD) remains problematic.

Resveratrol (RSV), a plant-derived compound, has also been shown to increase FAO flux in VLCADD patient cells [76]. Furthermore, RSV in combination with etomoxir, an inhibitor of CPT1 [77,78], has been investigated in human induced pluripotent stem cell (hiPSC) cardiomyocytes derived from VLCADD patients [77]. This resulted in improved mitochondrial energetics with a reduction in mitochondrial long-chain acylcarnitine accumulation, a feature which occurs in VLCADD [77]. This combined RSV-etomoxir treatment strategy warrants further investigation in human subjects; however, there may be complications with etomoxir treatment. A previous double-blind study of 350 patients was abandoned due to increased levels of liver transaminase in four patients taking etomoxir [79]. Furthermore, RSV has been shown to increase mitochondrial oxidative stress in mice [80], which may limit its potential as a therapeutic for VLCADD (although it should be noted that these experiments were in aged mice with weaker ability to defend oxidative stress). Indeed, elevated ROS generation has been reported for VLCADD (as will be discussed below), and as such ROS scavengers are also being investigated as a therapeutic approach [65].

Gene therapy using adeno-associated virus (AAV) has also been developed, with significant improvement in breathing with higher peak inspiratory flow and VLCAD protein expression in VLCAD-deficient mice [81]. Therapy with VLCAD mRNA-lipid nanoparticles was shown to improve respiration and carnitine accumulation in patient derived fibroblasts [82]. While a variety of treatment strategies are under investigation, most are focused on the primary VLCAD enzyme deficiency, and not on the potential associated mitochondrial metabolic defects, including OXPHOS dysfunction, which will be discussed below.

## 6. Mouse Models of VLCADD

Several different mouse models have been created to investigate the pathological mechanisms involved in VLCADD, including two different VLCAD^−/−^ mouse lines [83,84]. Interestingly, these VLCAD^−/−^ mice display a milder phenotype than human VLCADD, which may be explained by the compensatory function provided by LCAD in mice, which is largely absent in humans [85].

VLCAD^−/−^ mice show an accumulation of long-chain acylcarnitine species C16, C18, and C18:1, which is slightly different to that observed in human VLCADD (C14, C14:1, C14:2) [22,56,83,86]. VLCAD^−/−^ mice have normal viability and fecundity, as well as normal glucose levels after fasting [87]. However, they do exhibit exercise intolerance and develop cardiac hypertrophy [88,89,90]. VLCAD^−/−^ mice also exhibit elevated cold intolerance, resulting in bradycardia, hypothermia, and hypoglycemia, with increased lipid droplet accumulation in the liver and heart [87]. These mice also develop cardiomyopathy and metabolic decompensation under these stress conditions, which can be rescued by warming the mice, but not by glucose administration [87]. Cold stress also reduces the survival rate of VLCAD^−/−^ mice, which is due to the development of macrovascular hepatic steatosis [87].

As VLCAD and LCAD have overlapping functions in mice, LCAD^−/−^ mice have also been generated to model human long-chain fatty acid oxidation disorders. Interestingly, LCAD^−/−^ mice have a serum acylcarnitine profile that is similar to human VLCADD with a prominent accumulation of C14:1 [22,56,83]. LCAD^−/−^ mice have decreased fecundity, reduced litter sizes, exercise intolerance, and develop cardiac hypertrophy under normally-fed conditions [83,91]. They have heightened cold intolerance compared to VLCAD^−/−^ mice, with elevated hypothermia and hypoglycemia [92]. Overall, LCAD^−/−^ mice have a more severe phenotype than VLCAD^−/−^ mice, and as such may be a more suitable model for human VLCADD.

A double LCAD^−/−^/VLCAD^−/−^ mouse has also been generated, but is neonatally lethal [83,85], while an LCAD^−/−^/VLCAD^+/−^ mouse has a more sever phenotype compared to either the LCAD^−/−^ or VLCAD^−/−^ mouse models [85,93]. These LCAD^−/−^/VLCAD^+/−^ mice have aggravated hepatic steatosis and cardiac hypertrophy with a higher accumulation of acylcarnitine [93].

## 7. Interactions Between FAO and OXPHOS Proteins

While the processes of FAO and OXPHOS are clearly linked biochemically, there is growing evidence that physical interactions also exist between proteins involved in both pathways. Since the discovery of the first interaction between the FAO enzyme HADH and OXPHOS complex I, many more FAO-OXPHOS interactions have been described [94]. The electron transfer flavoprotein (ETF), which is responsible for electron shuttling between the FAO dehydrogenases and the OXPHOS complexes, was shown to physically associate with OXPHOS complex III [95]. Furthermore, OXPHOS supercomplexes have been shown to comigrate with VLCAD, LCAD, MCAD, ETF, and MTP using both sucrose gradient centrifugation and native gel-electrophoresis [10]. Using the sucrose gradient fractions containing high molecular mass OXPHOS supercomplexes, Wang et al. confirmed an association between these supercomplexes and FAO enzymes by showing that electron transfer through the OXPHOS complexes could be driven by fatty acids [10]. Further investigation has identified VLCAD (and other membrane ACADs) binding to OXPHOS complex I via an interaction with MTP [11] (Figure 1). More recently, HADHA (a subunit of the MTP) has been shown to be responsible for OXPHOS assembly in mouse cells, with knock down of HADHA expression resulting in reduced assembly and stability of the OXPHOS supercomplex [96]. Gel electrophoresis and co-immunoprecipitation studies confirmed interactions between HADHA and membrane components of the OPXHOS supercomplex, including complex I subunits [96]. HADHA knockdown also disrupted complex I assembly, with lipid droplet accumulation and impairment of mitochondrial oxygen consumption observed [96].

Other FAO proteins have been shown to perform specific roles in OXPHOS complex biogenesis, with the absence of ACAD9 leading to reduced assembly and steady-state levels of complex I [26]. While ACAD9 retains FAO dehydrogenase activity, it is essential for OXPHOS complex I biogenesis, suggesting that ACAD9′s primary role is in complex I assembly [26,97]. In addition to ACAD9, the FAO enzymes HADH and enoyl-CoA delta isomerase 1 (ECI1) are predicted to be involved in complex I assembly by phylogenetic comparison among different species that either have (or have lost) complex I structural subunit genes over time [98,99]. However, this putative assembly factor role for HADH and ECI1 has yet to be confirmed experimentally.

## 8. FAO Deficiency and Secondary OXPHOS Defects

Various FAO enzyme deficiencies have now been reported that are associated with secondary OXPHOS defects. Lactic acidosis, which denotes a defect in pyruvate oxidation due to potential OXPHOS dysfunction, was initially reported in long-chain 3-hydroxyacyl-CoA dehydrogenase deficiency (LCHADD) and VLCADD [100]. Following this, the first discovery of specific secondary OXPHOS defects was made in thirteen LCHADD patients, with a reduction in complex I, II, and III activities observed [101]. In addition to these OXPHOS defects, a concomitant increase in mitochondrial biogenesis (possibly as a compensatory mechanism) was also noted [101,102]. The complete absence of complex I, II, III, and IV activity in skeletal muscle from an LCHADD patient, and reduced activity of complex II and IV in patient cultured fibroblasts, have also been reported [103].

Animal studies have been performed to test whether the accumulation of long-chain fatty acid esters, as occurs in LCHAD, is toxic to OXPHOS function. Exposure of rat heart, skeletal muscle and brain mitochondria to specific fatty acid esters that accumulate in LCHADD caused an increase in mitochondrial oxygen consumption, altered reactive oxygen species balance, and mitochondrial permeability transition pore dysfunction [37,104,105].

Secondary OXPHOS defects have also been observed in MCADD, which may contribute to the neurological damage observed in some MCADD patients. Reduced oxygen consumption has been observed in both MCADD patient fibroblasts and MCAD ‘knock out’ (KO) cells, with reduced steady-state levels of the OXPHOS complexes detected [106]. Rat liver cells deficient in MCAD exhibit accumulation of medium-chain fatty acid esters (octanoate and decanoate), resulting in reduced OXPHOS complex I-III, II-III, and IV activities [107]. Similarly, rat muscle cells deficient in MCAD also showed a reduction in OXPHOS complex IV and II-III activities [107], with oxidative damage of membrane lipids and protein also detected [107]. Medium-chain length fatty acid toxicity is also associated with the disruption of mitochondrial calcium homeostasis, suggesting an additional MCADD pathogenic mechanism associated with calcium imbalance [108].

ECHS1 deficiency (ECHS1D) commonly present with Leigh Syndrome, a severe neurodegenerative condition characterized by bilateral symmetric brain lesions, developmental delay, dystonia, metabolic acidosis and cardiomyopathy [109]. Leigh Syndrome was originally associated with primary OXPHOS deficiencies and not FAOD; however, the first case of ECHS1D causing Leigh Syndrome was described in 2014 [110]. Subsequent analyses using ECHS1 KO cells lines and patient cell lines revealed reduced OXPHOS complex I and IV activity, with reduced steady-state levels of OXPHOS complex I, IV, and the I/III_2_/IV supercomplex, as well as reduced glucose and fatty acid oxidation [111]. Co-immunoprecipitation studies revealed the association of ECHS1 with subunits of OXPHOS complex I and V, as well as VLCAD [111].

In the reverse scenario, an OXPHOS complex I-deficient patient exhibited an abnormal blood acylcarnitine profile and enlarged mitochondria resembling LCHADD [112], with complex I-deficient patient fibroblasts exhibiting reduced VLCAD and ACAD9 protein expression [113]. Similarly, a deficiency in OXPHOS complex II was accompanied by abnormal acylcarnitine profiles in patient cells, with abnormal mitochondria structure observed using electron microscopy [114].

## 9. Relationship Between VLCADD and OXPHOS Defects

Clinically, there is strong evidence for associated mitochondrial dysfunction, including secondary OXPHOS defects, in primary VLCADD. Patient muscle biopsies with significant reduction in VLCAD activity show both OXPHOS complex I-III and II-III enzyme deficiencies [115]. Furthermore, mitochondria from VLCADD patients exhibit morphological defects under electron microscopy. Fewer mitochondria with disarrayed cristae, mitochondrial cysts, ruptured membranes, and large vacuoles have been described [116]. In addition, altered expression of the cellular proteome has been observed in VLCADD patients, with 10% of these proteins mitochondrially located [116]. ATPase downregulation and ion channel imbalance has also been reported in VLCADD patients with rhabdomyolysis [116].

VLCAD has been found to co-migrate with complex I and the OXPHOS supercomplex via native gel electrophoresis [10,11], with the loss of VLCAD expression resulting in the disappearance of specific OXPHOS supercomplex species in VLCAD^−/−^ mice [11]. VLCAD has also been shown to be physically associated with complex I and III in rat mitochondria via electron microscopy [11].

The toxic accumulation of fatty acyl-CoAs due to the loss of long-chain dehydrogenation has long been thought to be the main cause of OXPHOS enzyme dysfunction in primary FAOD. These toxic fatty acid esters can acylate essential intracellular proteins, including the OXPHOS enzyme complexes, leading metabolic dysfunction [117]. In addition, these fatty acid esters may cause a detergent-like effect, which would disrupt the lipid composition of the inner mitochondrial membrane [118]. This could result in destabilization of the OXPHOS complexes, which rely on specific membrane lipids for their stability (as observed in Barth syndrome where defects in inner membrane cardiolipin remodeling result in OXPHOS supercomplex destabilization) [119]. Indeed, reduced levels of cardiolipin have been identified in cases of MTP deficiency, which subsequently affect OXPHOS complex stability and function [120].

In support of the toxic fatty acid ester accumulation theory, blocking the entry and accumulation of fatty acyl-CoAs within mitochondria results in the restoration of electrophysiological dysfunction in VLCADD hiPSC-differentiated cardiomyocytes [77]. These cardiomyocytes exhibit shortened action potentials, delay after depolarization, increased Ca^2+^ concentration and long-chain acylcarnitine accumulation [77]. Blocking this accumulation with etomoxir normalized the action potential, reduced the frequency of delay after depolarization and stabilized Ca^2+^ concentrations [77]. Furthermore, rat skeletal muscle mitochondria incubated with fatty acids that specifically accumulate in VLCADD (cis-5-tetradecenoic acid and myristic acid) show inhibition of OXPHOS complex I-III activity (without affecting CII and CIV) [121]. Treatment with these two fatty acids also impairs oxygen consumption, ATP production, mitochondrial membrane potential, mitochondrial permeability transition pore opening, and mitochondrial calcium retention capacity [121]. Cis-5-tetradecenoic acid and myristic acid also decrease complex I (but not complex II) activity and mitochondrial membrane potential in rat heart mitochondria [122]. Moreover, state 4 (non-phosphorylating) respiration is increased, suggesting these two fatty acids may induce ETC uncoupling (however, membrane fluidity was not altered) [122].

VLCAD, along with other dehydrogenase enzymes, transfer electrons to OXPHOS complex III via the electron transfer flavoprotein (ETF) and the electron transfer flavoprotein–ubiquinone oxidoreductase (ETFDH) (Figure 1). This process is known to produce reactive oxygen species (ROS), with VLCAD activity resulting in direct production of H_2_O_2_ [123]. VLCADD patient fibroblasts cultured without glucose exhibit elevated ROS production associated with impairment of mitochondrial respiration and disruption of membrane permeability caused by Ca^2+^ imbalance [65]. Notably, normal function could be restored by treatment with ROS scavengers [65]. H_2_O_2_ can cause damage to mitochondrial inner membrane lipids via their peroxidation [124], which subsequently destabilizes the OXPHOS enzyme complexes, as described above [119]. Additionally, H_2_O_2_ can directly oxidize mitochondrial proteins, including OXPHOS complex structural subunits, which can also lead to OXPHOS dysfunction [113]. As such, VLCADD can increase ROS generation, with subsequent damage to mitochondrial inner membrane lipids that disrupt the stability and function of the OXPHOS complexes.

## 10. Conclusions

FAO is a crucial metabolic process, with deficiencies of the enzymes involved causing mitochondrial disease. VLCADD specifically culminates in FAO-dependent tissue pathology, such as skeletal muscle and heart disease, with the toxic accumulation of fatty acid esters playing a central role in the development of symptoms. Additionally, recent findings have highlighted the important interplay between members of the FAO and OXPHOS pathways, including interactions between VLCAD and the OXPHOS supercomplex. Loss of VLCAD expression can disrupt these important interactions, resulting in the possible destabilization of the OXPHOS complexes and their subsequent dysfunction. Indeed, there is significant evidence to show that secondary OXPHOS defects can occur in primary VLCADD. However, the specific FAO-OXPHOS interactions involved, and how the disruption of these interactions contributes to disease pathogenesis, remains largely unknown. Future investigation into these FAO-OXPHOS interactions will provide new insights into disease pathogenesis and pathways for the development of novel therapies that target both the primary FAO deficiency and the associated OXPHOS defects.

## Figures and Tables

**Table 1:** *ACADVL* mutations associated with an abnormal acylcarnitine profile suggesting VLCADD.

	Mutation	Protein	Reference	Presentation
1	c.65C>A	p.S22X	[52,53]	Symptomatic
2	c.134C>A	P.S45X	[52,53]	Symptomatic
3	c.494T>C	p.F165S	[52]	Symptomatic
4	c.1349G>A	p.R450H	[52,54,55]	Symptomatic
5	c.848T>C	p.V243A	[51,56,57]	Hepatic or Asymptomatic
6	c.1468G>C	p.A450P	[56,57]	Hepatic or Asymptomatic
7	c.602A>G	p.Y161C	[56]	Asymptomatic
8	c.865G>A	p.G289R	[56,58]	Asymptomatic
9	c.1376G>A	R419Q	[56]	Asymptomatic
10	c.1844G>A	p.R575Q	[51,57,58]	Cardiac
11	c.779C>T	p.T220M	[51,57]	Cardiac
12	c.1405C>T	p.R469W	[51,54,59]	Asymptomatic
13	c.1532G>C	R511P	[51,58]	Asymptomatic
14	c.1280G>A	W347ter Frame Shift	[57]	Hepatic
15	c.1600G>A	p.E454K	[57]	Sudden Death
16	c.1372T>C	p.F418L	[57]	Cardiac
17	c.739A>G	p.K207E	[57]	Sudden Death
18	G-1A	Splice site	[57]	Cardiac
19	A-2C	Splice site	[57]	Cardiac
20	Δ887-88	Frame Shift	[57]	Cardiac
21	c.1322G>A	p.G401D	[46,54,57,59]	Cardiac
22	c.637G>C	p.A173P	[57]	Sudden Death
23	G+1A	Splice site	[57]	Cardiac and Hepatic
24	Δ386-88	ΔE89 In Frame Deletion	[57]	Cardiac and Hepatic
25	ΔG-1	Splice site	[57]	Cardiac and Hepatic
26	c.1837C>T	p.R573W	[57,60]	Cardiac
27	41 bp insertion	Frame Shift	[57]	Cardiac
28	ΔG1621	Frame Shift	[57]	Cardiac and Hepatic
29	Δ891-3	ΔK258 In Frame deletion	[57]	Hepatic
30	ΔT932	Frame Shift	[57]	Cardiac
31	c.1146GNC	p.K382N	[58]	Asymptomatic
32	c.1076C>T	p.A359V	[58]	Asymptomatic
33	c.1504C>G	p.L502V	[58]	Asymptomatic
34	c.1066A>G	p.I356V	[58]	Asymptomatic
35	c.622G>A	p.G208R	[58]	Rhabdomyolysis
36	c.689C>T	p.T230I	[58]	Symptomatic
37	c.1173_1174insT	Frame Shift	[58]	Rhabdomyolysis
38	c.1806_1807delCT	Frame Shift	[58]	Hypoglycaemia
39	c.388_390delGAG	Unstable protein	[58]	Asymptomatic
40	c.439C>T	p.P147S	[58]	Elevated creatine kinase and liver function test
41	c.956C>A	stop codon	[58]	Elevated creatine kinase and liver function test
42	c.1001T>G	p.M334R	[58]	Asymptomatic
43	c.889-91delGAG	p.E297del	[54,61]	Cardiac and hypoglycaemia
44	c.1246G>T	p.A416S	[54,61]	Cardiac and hypoglycaemia
45	c.1097G>A	p.R366H	[54,59]	Elevated creatine kinase, rhabdomyolysis, metabolic acidosis, and hypoglycaemia
46	c.1019G>T	p.G340V	[54,62]	Asymptomatic
47	c.559A>G	p.K187E	[54]	Asymptomatic
48	c.1226C>T	p.T409M	[54,63]	Asymptomatic
49	c.481G>A	p.A161T	[54,64]	Mildly symptomatic
50	c.476A>G	p.Q159R	[54,59]	Mildly symptomatic
51	c.950T>C	p.V317A	[46,54,59]	Mildly symptomatic
52	c.1117A>T	p.I373F	[54,64]	Mildly symptomatic
53	c.1153C>T	p.R385W	[54,64]	Mildly symptomatic
54	c.1923G>C	p.L641P	[54]	Mildly symptomatic

## Data Availability

Not applicable.
